# Supplementary figures and images for: Social Influence and the Collective Dynamics of Opinion Formation
Source: PLoS One. 2013 Nov 5;8(11):e78433. doi: 10.1371/journal.pone.0078433 (PMC3818331; doi:10.1371/journal.pone.0078433)

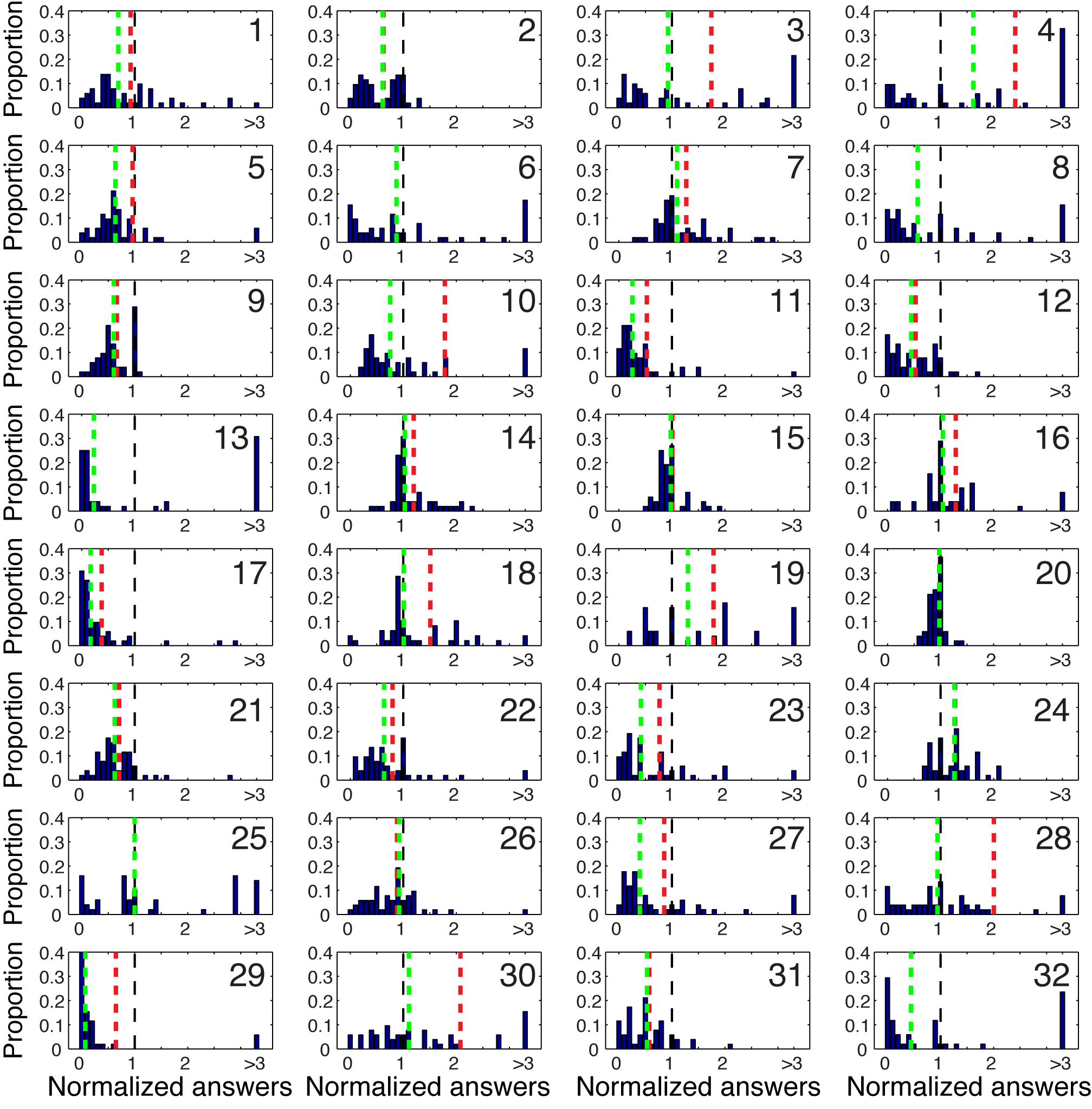

Supplement: Figure S1 — The distribution of answers for all 32 questions used in the first experiment (Experiment1, see Materials & Methods). The numbers on the upper right corner correspond to the question id, as indicated in the list of questions provided in the table S1. Question id = 27 has been used for illustrative purpose in the main text (Fig. 1A). The normalized answer is the estimate of the participants divided by the true value. The black dashed lines indicate the correct answer (normalized value = 1). The red and green dashed lines indicate the mean and the median values of the distribution, respectively. The mean values lying farther than 3 are not indicated. (TIF) [file pone.0078433.s001.tif]
