# Supplementary material for: Social Influence and the Collective Dynamics of Opinion Formation
Source: PLoS One. 2013 Nov 5;8(11):e78433. doi: 10.1371/journal.pone.0078433 (PMC3818331; doi:10.1371/journal.pone.0078433)
Supplement: Table S1 — Full list of questions used in the study. (DOCX) [file pone.0078433.s003.docx]

**Table S1**

| **Id** | **Question** | **Answer** |
| --- | --- | --- |
| **1** | *What is the length of the river Oder in kilometers?* | 866 |
| **2** | *What is the height of the Fernsehturm in Berlin (in meters)?* | 368 |
| ***3*** | *What is the height of Uluru (Ayers Rock) in Australia (in meters, ASL)?* | 863 |
| ***4*** | *How deep is the Baltic Sea at its deepest point (in meters)?* | 459 |
| ***5*** | *How long in the border between Germany and Switzerland (in km)?* | 316 |
| ***6*** | *What is the population density in Germany in inhabitants per square kilometer (2009)?* | 229 |
| ***7*** | *What is the distance between Berlin and London (in kilometers)?* | 910 |
| ***8*** | *How many inhabitants has the East Frisian island Wangerooge (retrieved 2010)?* | 919 |
| ***9*** | *How many members has the German Bundestag according to the law (without overhang seats; in 2012)?* | 598 |
| ***10*** | *How many universities are there in Germany (in 2010/2011)?* | 106 |
| ***11*** | *How many active nuclear power stations are there in Europe (retrieved 2011)?* | 196 |
| ***12*** | *How many countries take part in the general assembly of the United Nations as active members?* | 193 |
| ***13*** | *How many assaults per 100.000 inhabitants were officially registered in Germany in 2007?* | 618 |
| ***14*** | *What is the monthly amount of basic security benefits for full-aged, single job seekers (Hartz IV, in 2012)?* | 364 |
| ***15*** | *What is the monthly amount of child benefit in Germany for the first child (in 2012)?* | 184 |
| ***16*** | *How much does the iPad2 with 16GB cost (RRP)?* | 479 |
| ***17*** | *How many gold medals were awarded during the Olympics in China in 2008?* | 302 |
| ***18*** | *What is the world record in high jump of men (in centimeters)?* | 245 |
| ***19*** | *How long was the track for running events in a stadium in the Ancient Olympic Games (in meters)?* | 192 |
| ***20*** | *What is the maximum speed ever reached during a Formula One race (Grand Prix; in km/h)?* | 370 |
| ***21*** | *What is the world record in ski jumping of men (in meters)?* | 247 |
| ***22*** | *How long is the longest tee in golf so far (in meters)?* | 471 |
| ***23*** | *How many sportsmen have taken part in the first modern Olympic Games in Athens in 1896?* | 241 |
| ***24*** | *How many kilograms has a sumo wrestler to weight at least for being admitted as heavyweight?* | 115 |
| ***25*** | *What is the speed of sound in the air (MSL)?* | 343 |
| ***26*** | *How many bones does an adult human have?* | 206 |
| ***27*** | *What is the melting temperature of aluminum (in degrees Celsius)?* | 660 |
| ***28*** | *How many degrees Fahrenheit are 100 degrees Celsius?* | 212 |
| ***29*** | *How many earthquakes with a value of more than 6 on the Richter scale happen in an average year worldwide?* | 150 |
| ***30*** | *How many calories does one liter of Coca Cola contain (kcal)?* | 420 |
| ***31*** | *How many (earth)days has a year on the Mars?* | 687 |
| ***32*** | *How many times larger is the diameter of the sun compared to the diameter of the earth?* | 109 |
